# Association Between Immunoglobulin G *N*-glycosylation and Vascular Cognitive Impairment in a Sample With Atherosclerosis: A Case-Control Study

**DOI:** 10.3389/fnagi.2022.823468

**Published:** 2022-02-10

**Authors:** Meng Wang, Xueyu Chen, Zhaoyang Tang, Wenran Zhang, Haifeng Hou, Xiangfu Sun, Yuqing Shi, Xinxia Lu, Peirui Li, Long Ji, Guoyong Ding, Dong Li

**Affiliations:** ^1^Department of Epidemiology, School of Public Health, Shandong First Medical University & Shandong Academy of Medical Sciences, Tai’an, China; ^2^Department of Biostatistics, School of Public Health, Cheeloo College of Medicine, Shandong University, Jinan, China; ^3^Taian Traffic Hospital, Tai’an, China; ^4^The Second Affiliated Hospital of Shandong First Medical University, Tai’an, China

**Keywords:** atherosclerosis, vascular cognitive impairment, IgG *N*-glycans, inflammation, biomarker

## Abstract

**Background:**

Atherosclerosis is considered a crucial component in the pathogenesis of decreased cognitive function, as occurs in vascular cognitive impairment (VCI). Inflammation and the immune response play a significant role in the development of many chronic diseases. Immunoglobulin G (IgG) *N*-glycosylation has been implicated in the development of a variety of diseases by affecting the anti-inflammatory and proinflammatory responses of IgG. This study aimed to investigate the association between IgG *N*-glycosylation and VCI in a sample of patients with atherosclerosis through a case-control study.

**Method:**

We recruited a total of 330 patients with atherosclerosis to participate in this case-control study, including 165 VCI patients and 165 sex- and age-matched participants with normal cognitive function. The plasma IgG *N*-glycans of participants were separated by ultrahigh-performance liquid chromatography. An enzyme-linked immunosorbent assay (ELISA) kit was used to determine the corresponding serum inflammatory factors. Atherosclerosis was diagnosed by carotid ultrasound, and the diagnosis of VCI was based on the “Guidelines for the Diagnosis and Treatment of Vascular Cognitive Impairment in China (2019)”. A multivariate logistic regression model was used to explore the association between IgG *N*-glycans and VCI. We also analyzed the relationship between IgG *N*-glycans and the inflammatory state of VCI through canonical correlation analysis (CCA).

**Results:**

Through the multivariate logistic regression analysis, 8 glycans and 13 derived traits reflecting decreased sialylation and galactosylation and increased bisecting GlcNAc significantly differed between the case and control groups after adjusting for confounding factors (*P* < 0.05, *q* < 0.05). Similarly, the differences in TNF-α, IL-6, and IL-10 were statistically significant between the case and control groups after adjusting for the effects of confounding factors (*P* < 0.05, *q* < 0.05). The CCA results showed that VCI-related initial *N*-glycans were significantly correlated with VCI-related inflammatory factors (*r* = 0.272, *P* = 0.004). The combined AUC value (AUC_*combined*_ = 0.885) of 7 initial glycans and inflammatory factors was higher than their respective values (AUC_*initial glycans*_ = 0.818, AUC_*inflammatory factors*_ = 0.773).

**Conclusion:**

The findings indicate that decreased sialylation and galactosylation and increased bisecting GlcNAc reflected by IgG *N*-glycans might affect the occurrence of VCI in patients with atherosclerosis though promoting the proinflammatory function of IgG. IgG *N*-glycans may serve as potential biomarkers to distinguish VCI in individuals with atherosclerosis.

## Introduction

Vascular cognitive impairment (VCI) refers to any degree of cognitive impairment caused by cerebrovascular disease (van der Flier et al., 2018). VCI is second only to Alzheimer’s disease (AD) as the most common cause of dementia (Frances et al., 2016; Niu et al., 2019). The incidence of VCI is gradually increasing among elderly individuals worldwide, conferring an additional burden to individuals and society (Smith, 2017). The prevalence of VCI in the 65-year-old Chinese population is approximately 8.7%. Among the various cerebrovascular risk factors for VCI, atherosclerosis is an important cause of VCI (Arvanitakis et al., 2016; Jahrling et al., 2018; Hermkens et al., 2019). In addition, numerous studies have indicated that inflammation plays an important role in the progression of VCI (Aguilar-Navarro et al., 2016; Gregory et al., 2019) and atherosclerosis (Li et al., 2017). Inflammation is the basis of the pathological and physiological changes that occur in the process of atherosclerosis (Zhu et al., 2018); therefore, atherosclerosis is considered a chronic inflammatory disease (Ross, 1999). Atherosclerosis patients are prone to carotid artery narrowing and hemodynamic changes and reduced cerebral blood flow over time, thus increasing the risk of VCI (Simonetto et al., 2019). In addition, atherosclerosis is the main cause of cerebrovascular diseases (Sabeti et al., 2007; Vouillarmet et al., 2016), which undoubtedly increase the possibility of VCI development. Therefore, the discovery of biomarkers that might provide new ways to identify and prevent VCI is urgently needed for the population with atherosclerosis. Early identification of and timely intervention for VCI in individuals with atherosclerosis has positive clinical significance and value.

Glycosylation is an important modification of proteins that affects the structure and function of proteins. More than half of proteins have glycosylated structures (Kristic and Lauc, 2017). Immunoglobulin G (IgG) is the most abundant antibody in the human body and participates in the process of humoral immunity (Gudelj et al., 2018). The glycosylation of IgG can affect its effector functions, such as complement-dependent cytotoxicity (CDC) (Bohm et al., 2012) and antibody-dependent cell-mediated cytotoxicity (ADCC) (Masuda et al., 2007). The *N*-glycans attached to asparagine 297 in the CH_2_ domains of the fragment crystallization (Fc) region can change the anti-or proinflammatory functions of IgG (Alter et al., 2018). IgG exerts an anti-inflammatory effect when the bound *N*-glycan contains galactose, sialic acid or fucose, while the addition of bisecting *N*-acetylglucosamine (GlcNAc) has a proinflammatory effect (Kolarich et al., 2012; Wu et al., 2021). Previous studies have found that IgG *N*-glycosylation affects the development of many diseases (such as Parkinson’s disease (PD), AD, systemic lupus erythematosus, rheumatoid arthritis, cancer, and hyperuricemia) through its inflammatory role (Lundstrom et al., 2014; Vuckovic et al., 2015; Ren S. et al., 2016; Sebastian et al., 2016; Russell et al., 2017; Hou et al., 2019). At the same time, IgG *N*-glycosylation is also associated with risk factors for VCI, such as hypertension, dyslipidemia, ischemic stroke and type 2 diabetes (Wang et al., 2016; Lemmers et al., 2017; Liu et al., 2018a,b). We speculated that the occurrence and development of VCI may be associated with the inflammatory effect of IgG *N*-glycosylation.

In this study, we investigated the association between IgG *N*-glycans and VCI in patients with atherosclerosis, providing new and possible biomarkers for the screening of VCI. In addition, we explored the association between IgG *N*-glycans and inflammatory factors, which may further explain the role of IgG *N*-glycosylation in the process of VCI in patients with atherosclerosis.

## Materials and Methods

### Study Sample

In this case-control study, a total of 330 participants with atherosclerosis were recruited from the Taian Traffic Hospital and the Jidong Oil-field Hospital of Chinese National Petroleum in 2019. The 330 participants included 165 VCI patients (case group) and 165 sex- and age-matched participants with normal cognitive function (control group). The inclusion criteria of all participants were as follows: (1) Chinese Han ethnicity, (2) age 60 years or older, and (3) a diagnosis of atherosclerosis. The exclusion criteria were as follows: (1) a diagnosis of a serious disease (such as cancer, chronic obstructive pulmonary disease, etc.); (2) a diagnosis of an autoimmune disease (such as systemic lupus erythematosus, rheumatoid arthritis, etc.); and (3) a history of depression or mental illness. This study was approved by the Ethics Committee of Shandong First Medical University and conducted according to the guidelines of the Declaration of Helsinki. Written informed consent was obtained from all participants before the study.

### Diagnosis of Atherosclerosis and Vascular Cognitive Impairment

#### Diagnosis of Atherosclerosis

A color Doppler ultrasound diagnostic apparatus (SSD-1700; Aloka, Japan) was used to examine participants in a supine posture for the presence of atherosclerosis. The frequency of the ultrasound probe was 5-10 MHz. On both sides of the common carotid arteries, the carotid bifurcation, external carotid artery and internal carotid artery were examined in turn, and the intima-media thickness (IMT) value of the tube wall was recorded. Atherosclerosis was defined as increased IMT or plaques. An IMT ≥ 1 mm was defined as an increased IMT, and a plaque was defined as an area with an IMT ≥ 1.5 mm (Wang et al., 2018). In our study, physicians recorded atherosclerosis directly from IMT findings based on diagnostic criteria.

#### Diagnosis of Vascular Cognitive Impairment

The diagnosis of VCI in this study was based on the “Guidelines for the Diagnosis and Treatment of Vascular Cognitive Impairment in China (2019)”. The diagnosis of VCI mainly included three aspects: (1) a Mini-Mental State Examination (MMSE) score (classified as ≤ 17 (illiterate), ≤ 20 (primary school), ≤ 24 [junior high school or high school), or ≤ 27 (college/university and above)] confirming the presence of cognitive impairment (Liu et al., 2017); (2) the presence of one or more cerebrovascular diseases or vascular injury lesions, which was confirmed using magnetic resonance imaging (MRI) or computed tomography (CT) (Niu et al., 2019), and the diagnosis of imaging findings required at least one of the following: ① a diagnosis of a large-vessel cerebral infarction; ② a diagnosis of an extensive or critical cerebral infarction; ③ a diagnosis of extensive or fused white matter hyperintensity; ④ a diagnosis of two or more lacunar infarctions other than the brainstem; ⑤ a diagnosis of one to two lacunas in critical parts, or one to two lacunas in non-critical parts with extensive white matter hyperintensity; and ⑥ a diagnosis of cerebral hemorrhage in critical parts, or two or more cerebral hemorrhages; and (3) the occurrence of cognitive impairment was temporally related to one or more cerebrovascular events. All diagnoses were performed independently by two physicians based on imaging changes and clinical manifestations. Participants with all three of these aspects were diagnosed with VCI, and were included in the case group. In order to distinguish between the case group and the control group, all the participants performed the above diagnostic procedure.

### Measurement of Inflammatory Factors and IgG *N*-glycans

Previous studies have showed that high-sensitivity C-reactive protein (hs-CRP), tumor necrosis factor-alpha (TNF-α) and interleukin-6 (IL-6) are related to VCI, interleukin-4 (IL-4) is important to brain repairing, and interleukin-10 (IL-10) is essential for maintaining normal neuroimmune communication and related to the severity of atherosclerosis (Mallat et al., 1999; Richwine et al., 2009; Miralbell et al., 2013; Guoping et al., 2015; Liu et al., 2016; Chua et al., 2020). Therefore, CRP, TNF-a, IL-6, IL-10, and IL-4 were selected to the index of inflammatory factors. The levels of TNF-α, hs-CRP, IL-4, IL-6, and IL-10 were determined using the relevant enzyme-linked immunosorbent assay (ELISA) kit (Chang et al., 2016).

The measurement method of IgG *N*-glycans was as previously reported (Liu et al., 2020). Briefly, the method mainly consisted of four steps: (1) IgG was isolated from a plasma sample using a protein G monolithic plate (CIM^®^ r-Protein G 0.2ml Monolithic 96-well Plate, BIA Separations, Slovenia) (Pucic et al., 2011); (2) the isolated IgG sample released IgG *N*-glycans following the addition of PNGase F enzyme; (3) the released IgG *N*-glycans were labeled with 2-aminobenzamide (2-AB) mixed solution reagent, and then the labeled IgG *N*-glycans were purified using filter plates (AcroPrep Advance 96-well Filter Plates -1 mL, 0.2 μm wwPTFE membrane, Pall Corporation, San Diego, CA, United States); and (4) IgG *N*-glycans were detected by hydrophilic interaction chromatography (HILIC)-ultra-performance liquid chromatography (UPLC) (Walters Corporation, Milford, MA, United States). In the end, IgG *N*-glycans were identified as 24 glycan peaks (GPs 1-24 or 24 initial glycans), a chromatogram showing the individual differences between the case group and the control group is shown in Supplementary Figure 1, and the glycan structures in each peak are shown in Supplementary Figure 2. GP3 was excluded from all calculations because it did not pass the quality control standards (Novokmet et al., 2014), because GP3 was coeluted with contaminants in some samples, which significantly affected its value (Yu et al., 2016). In addition, 17 derived traits were calculated using initial glycans to represent the relative abundances of core fucosylation, sialylation, bisecting GlcNAc, and galactosylation (Ren S. et al., 2016; Qin et al., 2019; Table 1).

**TABLE 1:** The calculation formula of derived glycans.

Derived traits	Description	Computational method
GPN	Proportion of neutral glycans in total IgG glycans	GP1 + GP2 + GP4 + GP5 + GP6 + GP7 + GP8 + GP9 + GP10 + GP11 + GP12 + GP13 + GP14 + GP15
S_total_	Proportion of sialylated glycans in total IgG glycans	GP16 + GP17 + GP18 + GP19 + GP21 + GP22 + GP23 + GP24
S1	Proportion of monosialylated glycans in total IgG glycans	GP16 + GP17 + GP18 + GP19
S2	Proportion of disialylated glycans in total IgG glycans	GP21 + GP22 + GP23 + GP24
G0	Proportion of agalactosylated glycans in total IgG glycans	GP1 + GP2 + GP4 + GP6
G1	Proportion of monogalactosylated glycans in total IgG glycans	GP7 + GP8 + GP9 + GP10 + GP11
G2	Proportion of galactosylated glycans in total IgG glycans	GP12 + GP13 + GP14 + GP15
F	Proportion of fucosylated glycans in total IgG glycans	GP1 + GP4 + GP6 + GP8 + GP9 + GP10 + GP11 + GP14 + GP15 + GP16 + GP18 + GP19 + GP23 + GP24
FN	Proportion of fucosylated glycans in total neutral IgG glycans	(GP1 + GP4 + GP6 + GP8 + GP9 + GP10 + GP11 + GP14 + GP15)/GPN*100
FS	Proportion of fucosylated glycans in total sialylated IgG glycans	(GP16 + GP18 + GP19 + GP23 + GP24)/S_*total*_ *100
B	Proportion of bisecting glycans in total IgG glycans	GP6 + GP10 + GP11 + GP13 + GP15 + GP19 + GP22 + GP24
BN	Proportion of bisecting glycans in neutral IgG glycans	(GP6 + GP10 + GP11 + GP13)/GPN*100
BS	Proportion of bisecting glycans in sialylated IgG glycans	(GP19 + GP22 + GP24)/S_*total*_ *100
FG0	Proportion of fucosylated agalactosylated glycans in total IgG glycans	GP4
FG1	Proportion of fucosylated monogalactosylated glycans in total IgG glycans	GP8 + GP9
FG2	Proportion of fucosylated galactosylated glycans in total IgG glycans	GP14
aGal/Gal ratio	the relative intensities of agalactosylated (G0) *vs* monogalactosyl (G1) and digalactosyl (G2) *N*-glycans	G0/(G1 + G2*2)*100

*B, bisecting GlcNAc; F, core fucose; G, galactose; N, neutral glycans; S, sialic acid.*

### Assessment of Covariates

The uniformly trained investigators used questionnaires to collect demographic characteristics such as the age, sex, education level, and income of participants. Body mass index (BMI) was calculated by measuring height and weight, and the calculation formula was weight (kg)/height^2^ (m^2^).

A blood sample was collected from each participant’s large antecubital vein through venipuncture in the morning after overnight fasting. The sample was collected into two tubes: the sample collected in a vacuum tube containing ethylenediaminetetraacetic acid (EDTA) underwent plasma separation to detect IgG *N*-glycans, while the sample collected in a tube without EDTA underwent serum separation to determine inflammatory factors and blood biochemical indexes.

Hypertension was defined as a systolic blood pressure (SBP) ≥140 mmHg and/or diastolic blood pressure (DBP) ≥90 mmHg. Diabetes mellitus was defined as a fasting blood glucose level ≥7.0 mmol/L (126 mg/dL) (Cruickshank, 2009). According to the guidelines for the prevention and control of dyslipidemia in adults in China, dyslipidemia was defined as high-density lipoprotein cholesterol (HDL-C) <1.0 mmol/L, low-density lipoprotein cholesterol (LDL-C) ≥4.1 mmol/L, triglycerides (TGs) ≥2.3 mmol/L, or total cholesterol (TC) ≥6.2 mmol/L (Wang et al., 2011).

### Statistical Analyses

The Kolmogorov-Smirnov test was used to check the normal distribution of variables. Continuous variables with a normal distribution are represented as the mean ± standard deviation (SD) and were compared with an independent-sample *t* test. Continuous variables with a non-normal distribution were represented as the median (*P*_25_-*P*_75_) and were compared with the Wilcoxon rank-sum test. Categorical data are represented as n (%) and were compared with the chi-square test.

Multivariate logistic regression analyses were used to determine the association between VCI and each of 23 initial glycans, 17 derived traits and inflammatory factors after adjusting for the effects of age, BMI, education, income, smoking, drinking, salt intake habit, hypertension, hyperlipidemia, and diabetes mellitus. For the multiple corrections, the false discovery rate (FDR) was used based on the Benjamini–Hochberg procedure (*q*). Spearman correlation analysis was used to calculate the correlation coefficient (*r*_*s*_) between initial glycans. Canonical correlation analysis (CCA) was used to explore the relationship between VCI-related initial glycans and VCI-related inflammatory factors and to obtain the overall correlation between the two sets. The initial glycans (or inflammatory factors) associated with VCI were included in stepwise multivariate logistic regression analyses to screen glycan biomarkers (or inflammation biomarkers) for VCI diagnosis after adjusting for the above confounding factors. Then, receiver operating characteristic (ROC) curve analysis was applied to calculate the area under the curve (AUC) to evaluate the classification performance of glycan biomarkers, inflammation biomarkers and the combination of the two to distinguish VCI.

The statistical analyses were performed using SPSS 25.0 (IBM, Armonk, NY, United States), SAS software, version 9.4 (SAS Institute Inc., Cary, NC, United States) and R version 3.4.3 (R Core Team). All statistical tests were two-sided. The *q* value represented the *P* value after correction for multiple testing, statistical significance was defined as *P* < 0.05 and *q* < 0.05.

## Results

### Baseline Characteristics of the Study Participants

A total of 330 patients with atherosclerosis were recruited to participate in this case-control study. The 330 participants included 165 VCI patients (case group, 79 men/86 women, mean age 66.46 ± 3.83 years) and 165 sex- and age-matched participants with normal cognitive function (control group). The basic characteristics of the participants in the two groups are summarized and compared in Table 2. Significant differences in education (*P* = 0.001), income (*P* = 0.005), hypertension (*P* = 0.012), SBP (*P* = 0.014), and TG (*P* = 0.029) were found between the groups.

**TABLE 2:** Characteristics of the study participants.

Characteristics	Cases (*n* = 165)	Controls (*n* = 165)	*t*/χ^2^	*P*
Gender (male/female)	79/86	79/86	—	—
Age (years)	66.46 ± 3.83	66.26 ± 3.74	0.480	0.632
BMI (kg/m^2^)	24.91 ± 3.10	24.59 ± 3.12	0.941	0.348
Education (*n*, %)			14.709	0.001*
Illiteracy/primary school	46 (27.88)	19 (11.51)		
Middle school	101 (61.21)	118 (71.52)		
College/university	18 (10.91)	28 (16.97)		
Income, ¥/month (*n*, %)			10.692	0.005*
≤ ¥3000	63 (38.18)	41 (24.85)		
¥3001-4999	95 (57.58)	105 (63.64)		
≥ ¥5000	7 (4.24)	19 (11.51)		
High salt intake (*n*, %)	43 (26.06)	29 (17.58)	3.377	0.066
Smoking (*n*, %)	25 (15.15)	24 (14.55)	0.024	0.877
Drinking (*n*, %)	25 (15.15)	29 (17.58)	0.416	0.519
Hypertension (*n*, %)	73 (44.24)	51 (30.91)	6.253	0.012*
Dyslipidemia (*n*, %)	91 (55.15)	82 (49.70)	0.984	0.321
Diabetes (*n*, %)	28 (16.97)	26 (15.76)	0.089	0.766
SBP (mmHg)	136.67 ± 17.96	132.01 ± 16.23	2.473	0.014*
DBP (mmHg)	81.70 ± 11.06	80.42 ± 10.71	1.072	0.284
GLU (mmol/L)	5.66 ± 0.84	5.63 ± 1.04	0.328	0.743
TG (mmol/L)	1.86 ± 1.43	1.58 ± 0.80	2.196	0.029*
TC (mmol/L)	5.25 ± 1.05	5.18 ± 1.04	0.607	0.544
HDL-C (mmol/L)	1.21 ± 0.22	1.22 ± 0.24	0.319	0.750
LDL-C (mmol/L)	2.39 ± 0.68	2.32 ± 0.70	0.897	0.370
hs-CRP (mg/L)	3.31 ± 1.12	3.19 ± 1.08	0.982	0.327
TNF-α (pg/mL)	23.39 ± 5.78	19.29 ± 5.88	6.386	<0.001*
IL-4 (pg/mL)	10.76 ± 2.87	10.45 ± 2.86	1.000	0.318
IL-6 (pg/mL)	6.64 ± 2.34	6.02 ± 2.65	2.248	0.025*
IL-10 (pg/mL)	133.73 ± 23.75	159.43 ± 46.29	6.343	<0.001*

*Statistically significant, P<0.05. P values were calculated by the independent-sample t-test or chi-square test, as appropriate. BMI, body mass index; DBP, diastolic blood pressure; GLU, glucose; HDL-C, high density lipoprotein cholesterol; hs-CRP, high-sensitivity C-reactive protein; IL-4, interleukin-4; IL-6, interleukin-6; IL-10, interleukin-10; LDL-C, low density lipoprotein cholesterol; SBP, systolic blood pressure; TC, total cholesterol; TG, total triglycerides; TNF-α, tumor necrosis factor-alpha.

### Association of Inflammatory Factors With Vascular Cognitive Impairment

**FIGURE 1:**
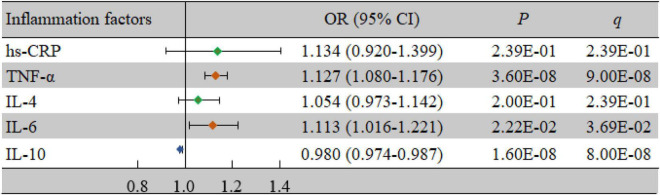
Associations of inflammatory factors and VCI as determined by multivariate logistic regression analyses. *P* < 0.05 was considered statistically significant using logistic regression analysis; *q* < 0.05: significant after correction using FDR. The multivariate logistic regression analyses were performed after adjusting for age, BMI, education, income, smoking, drinking, salt intake habit, hypertension, hyperlipidemia, and diabetes mellitus. BMI, body mass index; CI, confidence interval; FDR, false discovery rate; hs-CRP, high-sensitivity C-reactive protein; IL-4, interleukin-4; IL-6, interleukin-6; IL-10, interleukin-10; OR, odds ratio; TNF-α, tumor necrosis factor-alpha; VCI, vascular cognitive impairment.

As shown in Table 2, the levels of TNF-α (*P* < 0.001) and IL-6 (*P* = 0.025) in the case group were significantly higher than those in the control group, while the level of IL-10 (*P* < 0.001) was significantly lower in the case group than in the control group (*P* < 0.05). At the same time, TNF-α (odds ratio (OR) = 1.127, 95% confidence interval (CI): 1.080-1.176, *P* < 0.001, *q* < 0.001), IL-6 (OR = 1.113, 95% CI: 1.016-1.221, *P* = 0.022, *q* = 0.037), and IL-10 (OR = 0.980, 95% CI: 0.974-0.987, *P* < 0.001, *q* < 0.001) were significantly associated with VCI after adjusting for confounding factors (Figure 1).

### Association of IgG *N*-glycans With Vascular Cognitive Impairment

**FIGURE 2:**
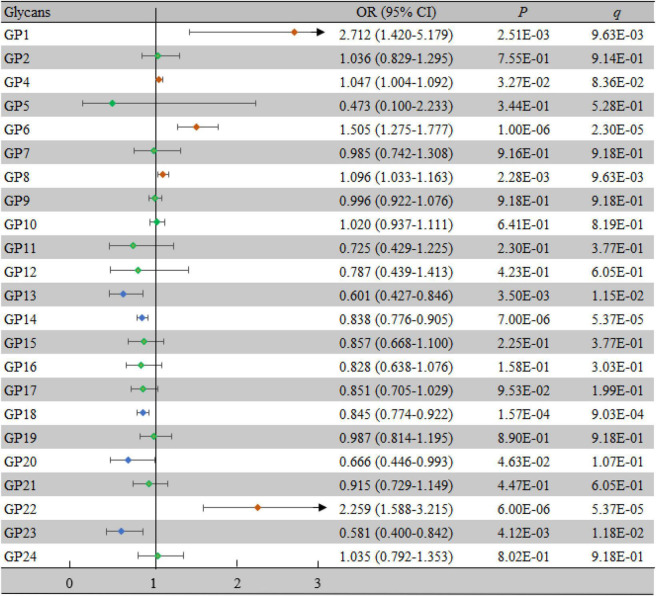
Associations of the normalized initial glycans and VCI as determined by multivariate logistic regression analyses. *P* < 0.05 was considered statistically significant using logistic regression analysis; *q* < 0.05: significant after correction using FDR. The multivariate logistic regression analyses were performed after adjusting for age, BMI, education, income, smoking, drinking, salt intake habit, hypertension, hyperlipidemia, and diabetes mellitus. BMI, body mass index; CI, confidence interval; FDR, false discovery rate; GP, glycan peak; OR, odds ratio; VCI, vascular cognitive impairment.

Comparisons of the levels of 23 initial glycans between the case group and the control group are shown in Supplementary Table 1, while comparisons of the 17 derived traits are shown in Supplementary Table 2. There were significant differences in 8 initial glycans and 14 derived traits between the two groups (all *P* < 0.05, all *q* < 0.05). Furthermore, we identified whether each IgG *N*-glycan was associated with VCI by multivariate logistic regression analyses. As shown in Figure 2, 8 initial glycans (reduced relative abundance of GP13, GP14, GP18, and GP23 as well as increased relative abundance of GP1, GP6, GP8, and GP22) were significantly associated with VCI after adjusting for the effects of confounding factors (all *P* < 0.05, all *q* < 0.05).

**FIGURE 3:**
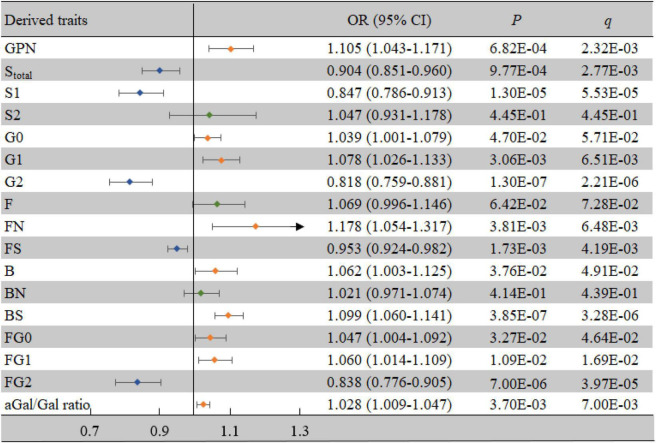
Associations of the derived traits and VCI as determined by multivariate logistic regression analyses. *P* < 0.05 was considered statistically significant using logistic regression analysis; *q* < 0.05: significant after correction using FDR. The multivariate logistic regression analyses were performed after adjusting for age, BMI, education, income, smoking, drinking, salt intake habit, hypertension, hyperlipidemia, and diabetes mellitus. B, bisecting GlcNAc; BMI, body mass index; CI, confidence interval; F, core fucose; FDR, false discovery rate; G, galactose; N, neutral glycans; OR, odds ratio; S, sialic acid; VCI, vascular cognitive impairment.

For the derived traits, 13 derived traits in the IgG *N*-glycans were significantly associated with VCI after adjusting for confounding factors (all *P* < 0.05, all *q* < 0.05) (Figure 3), which mainly reflected the decrease in sialylation (S_*total*_, OR = 0.904, 95% CI: 0.851-0.960, *P* = 0.001, *q* = 0.003; S1, OR = 0.847, 95% CI: 0.786-0.913, *P* < 0.001, *q* < 0.001) and galactosylation (G2, OR = 0.818, 95% CI: 0.759-0.881, *P* < 0.001, *q* < 0.001; aGal/Gal ratio, OR = 1.028, 95% CI: 1.009-1.047, *P* = 0.004, *q* = 0.007), as well as the increase in neutral *N*-glycans (GPN, OR = 1.105, 95% CI: 1.043-1.171, *P* = 0.001, *q* = 0.002) and bisected GlcNAc (B, OR = 1.062, 95% CI: 1.003-1.125, *P* = 0.038, *q* = 0.049).

### Correlation Between Vascular Cognitive Impairment-Related Inflammatory Factors and Vascular Cognitive Impairment-Related IgG *N*-glycans

**FIGURE 4:**
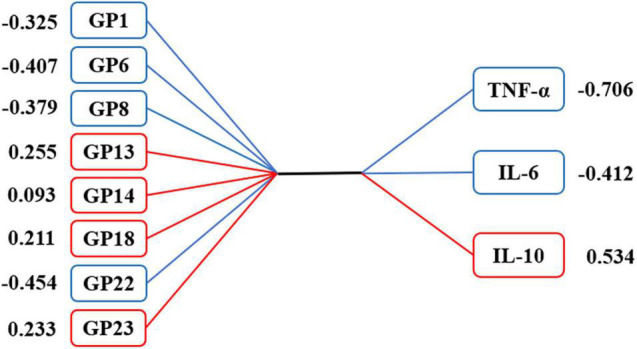
Canonical structures of initial glycans and inflammation markers in the first canonical set of canonical correlation analysis. The absolute value of canonical loadings greater than 0.300 was a significant loading. The positive relationships are shown in red boxes, while negative relationships are shown in blue boxes. GP, glycan peak; hs-CRP, high-sensitivity C-reactive protein; IL-4, interleukin-4; IL-6, interleukin-6; IL-10, interleukin-10; TNF-α, tumor necrosis factor-alpha.

The CCA results showed that VCI-related IgG *N*-glycans were significantly correlated with VCI-related inflammatory factors in the first canonical set, and the canonical correlation coefficient was 0.272 (*P* = 0.004, Supplementary Table 3). As shown in Figure 4, 4 initial traits (GP1, GP6, GP8, and GP22) tended to be significantly associated with TNF-α, IL-6, and IL-10 levels. In addition, a strong association was observed between GP22 and canonical variables, with a loading of −0.454, and the response variable with the highest canonical loading was −0.706 (TNF-α).

### Classification of Vascular Cognitive Impairment Using IgG *N*-glycans and Inflammation Markers

**FIGURE 5:**
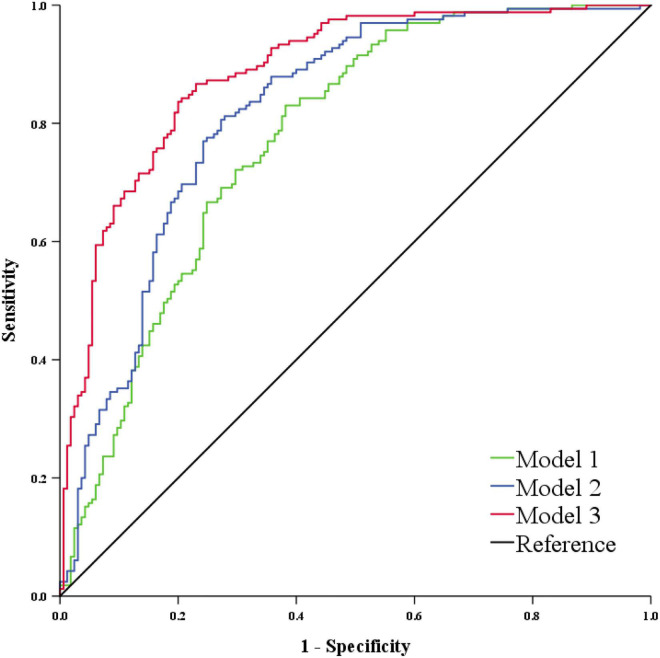
ROC curve analysis in regard to the binary logistic regression model for the prediction of VCI. Model 1 consists of GP1, GP6, GP8, GP13, GP14, GP22, and GP23. Model 2 consists of TNF-α, IL-6, and IL-10. Model 3 consists of Model 1 and Model 2. GP, glycan peak; IL-6, interleukin-6; IL-10, interleukin-10; ROC, receiver operator characteristic; TNF-α, tumor necrosis factor-alpha; VCI, vascular cognitive impairment.

As shown in Supplementary Figure 3, there were significant correlations among the 8 VCI-related initial glycans. Therefore, we performed a stepwise multivariate logistic regression analysis on the above 8 initial glycans to avoid the influence of collinearity and to select biomarkers for the diagnosis of VCI. Finally, 7 initial glycans were selected to establish a classification model to distinguish the case group from the control group (Supplementary Table 4), and the AUC value of the model including these 7 initial glycans (GP1, GP6, GP8, GP13, GP14, GP22, and GP23) was determined to be 0.818 (95% CI: 0.772-0.864). Similarly, TNF-α, IL-6, and IL-10 were selected as inflammation markers for the diagnosis of VCI through a stepwise multivariate logistic regression analysis (Supplementary Table 5), and the AUC value of the model consisting of TNF-α, IL-6, and IL-10 was determined to be 0.773 (95% CI: 0.723-0.824). In addition, we found that the combined AUC value of the 7 initial glycans and inflammation biomarkers was higher than their respective values, with an AUC of 0.885 (95% CI: 0.849-0.921) (Figure 5).

## Discussion

In our study, we found that low levels of galactosylation and sialylation and increased levels of bisecting GlcNAc reflected by IgG *N*-glycans in individuals with atherosclerosis may increase the risk of VCI. At the same time, IgG *N*-glycans were associated with the changes in inflammatory factors observed in the study.

Our research found that abnormal IgG *N*-glycosylation (decreased galactosylation and sialylation and increased bisecting GlcNAc) in individuals with atherosclerosis might increase the risk of VCI, which is consistent with previous studies in AD. A study on AD and IgG *N*-glycosylation found that the levels of galactosylation and sialylation in the plasma of patients with AD were lower than those in the healthy control group (Lundstrom et al., 2014). In cerebrospinal fluid samples from patients with AD, abnormal *N*-glycosylation was found, including decreased sialylation and increased bisecting GlcNAc compared with samples from individuals with mild cognitive impairment (MCI) (Palmigiano et al., 2016). A reduction in sialylation in IgG *N*-glycans was reported to be associated with PD, and PD patients had higher GP8 levels, which was consistent with our conclusions (Russell et al., 2017). This evidence fully demonstrated that abnormal IgG *N*-glycosylation was closely related to neurodegenerative diseases. A possible explanation for this relationship is that abnormal IgG *N*-glycosylation could alter the inflammatory effect of IgG, causing it to switch from anti-inflammatory to proinflammatory functions to participate in the inflammatory response (Novokmet et al., 2014; de Jong et al., 2016), and inflammation is also an important factor affecting neurodegenerative diseases such as VCI, AD, and PD (Heppner et al., 2015; Pajares et al., 2020; Wang et al., 2020).

In this study, high levels of proinflammatory factors (TNF-α, IL-6) and low levels of an anti-inflammatory factor (IL-10) were found in the case group, which indicates that inflammation might be one of the characteristics of VCI. This possibility is supported by the fact that inflammation is a known risk factor for VCI (Aguilar-Navarro et al., 2016; Wang et al., 2020). The decreased level of IL-10 in the study might be caused by the high expression of TNF-α (Ren Z. Q. et al., 2016). The inflammatory response and oxidative stress regulated by inflammatory factors are considered to be the key factors in cerebrovascular diseases (Iadecola, 2013; Kiss et al., 2019). Under the action of inflammation and oxidative stress, continuous vascular damage destroys neurovascular units, leading to the destruction of blood-brain barrier permeability, aggravating tissue hypoxia, and damaging neurons and white matter, thereby resulting in VCI (Carrano et al., 2011; Gorelick et al., 2016). Atherosclerosis is also regulated by inflammation, and intensified inflammation activation promotes the progression of atherosclerosis, which may lead to thrombus formation and plaque rupture and ultimately increase the risk of VCI (Hansson et al., 2006; Seneviratne and Monaco, 2015).

We found that there was an overall correlation between VCI-related initial glycans and VCI-related inflammatory factors. The structure of these initial glycans corresponds to sialylation, galactosylation, and bisecting GlcNAc of IgG *N*-glycans. Due to the low level of galactosylation and sialylation and the high level of bisecting GlcNAc in IgG *N*-glycans, IgG has a proinflammatory effect (Kolarich et al., 2012; Wu et al., 2021). The reduction of the sialylation level of IgG increases the affinity of IgG to the Fcγ-receptor IIIa (Fcγ-RIIIa), up-regulates the ADCC pathway, and promotes inflammatory activity (Bohm et al., 2012). The high level of bisecting GlcNAc in IgG *N*-glycans can also enhance the ADCC effect of IgG. The reduction of galactosylation inhibits the binding affinity of IgG to complement component 1q (C1q) and inhibits the CDC effect, thereby enhancing the pro-inflammatory function (Zou et al., 2011; Peschke et al., 2017). Therefore, the inflammatory state of IgG affected by IgG *N*-glycosylation might partially explain the inflammation that accompanies the development of VCI in individuals with atherosclerosis.

This study has several strengths. To the best of our knowledge, this is the first study to explore the association between IgG *N*-glycans and VCI in a sample of patients with atherosclerosis. We also analyzed the relationship between IgG *N*-glycans and inflammatory factors, and the findings helped to explain the effect of IgG *N*-glycosylation on VCI. However, there are some limitations in our study. First, this study was a case-control study, and it was difficult to infer the chronological and causal relationship between IgG *N*-glycosylation and VCI. Second, in terms of identifying MCI, Montreal Cognitive Assessment (MoCA) might be a better screening tool than MMSE, but both tests were found to be accurate in the detection of AD. MMSE is still one of the most widely used cognitive screening tests in the world (Pinto et al., 2019). It was difficult to classify the degree of VCI, such as subjective cognitive impairment, MCI, and dementia, according to the existing data in this study. At the same time, this study did not further group discussions on the degree of atherosclerosis based on the IMT value due to data limitations. In addition, the sample included in this study was small, and only one ethnic group (Chinese Han) was included. Therefore, it is necessary to verify this hypothesis in studies with large sample sizes and multiethnic samples and to use cohort studies or Mendelian randomization studies to accurately explore the relationship between IgG *N*-glycosylation and VCI in individuals with atherosclerosis.

## Conclusion

In summary, the results from our study indicated that IgG *N*-glycans might be related to the inflammation that accompanies the development of VCI in individuals with atherosclerosis. The decreased sialylation and galactosylation and the increased bisecting GlcNAc reflected by the IgG *N*-glycans of VCI patients might affect the occurrence of VCI by changing the inflammatory effect of IgG. Moreover, IgG *N*-glycans may serve as potential biomarkers to distinguish VCI in individuals with atherosclerosis and, when combined with inflammatory factors, improve the ability to diagnose VCI.

## Data Availability Statement

The datasets presented in this study can be found in online repositories. The names of the repository/repositories and accession number(s) can be found in the article/Supplementary Material.

## Ethics Statement

This study was approved by the Ethics Committee of Shandong First Medical University and conducted according to the guidelines of the Declaration of Helsinki. The patients/participants provided their written informed consent to participate in this study.

## Author Contributions

DL and LJ conceived the whole research and initiated the project, supervised the overall project design and execution. MW, XC, and GD designed all the experiments and wrote the manuscript. ZT, WZ, HH, and XS participated in data analysis and interpretation. YS, XL, and PL helped us collect data and analyze structure. All authors contributed to the article and approved the submitted version.

## Conflict of Interest

The authors declare that the research was conducted in the absence of any commercial or financial relationships that could be construed as a potential conflict of interest.

## Publisher’s Note

All claims expressed in this article are solely those of the authors and do not necessarily represent those of their affiliated organizations, or those of the publisher, the editors and the reviewers. Any product that may be evaluated in this article, or claim that may be made by its manufacturer, is not guaranteed or endorsed by the publisher.

## References

[B1] Aguilar-Navarro S. G. Mimenza-Alvarado A. J. Anaya-Escamilla A. Gutierrez-Robledo L. M. (2016). Frailty and vascular cognitive impairment: mechanisms behind the link. *Rev. Invest. Clin.* 68 25–32. 10.1007/978-981-10-1433-8_3 27028174

[B2] Alter G. Ottenhoff T. H. M. Joosten S. A. (2018). Antibody glycosylation in inflammation, disease and vaccination. *Semin. Immunol.* 39 102–110. 10.1016/j.smim.2018.05.003 29903548 PMC8731230

[B3] Arvanitakis Z. Capuano A. W. Leurgans S. E. Bennett D. A. Schneider J. A. (2016). Relation of cerebral vessel disease to Alzheimer’s disease dementia and cognitive function in elderly people: a cross-sectional study. *Lancet Neurol.* 15 934–943. 10.1016/S1474-4422(16)30029-1 27312738 PMC4969105

[B4] Bohm S. Schwab I. Lux A. Nimmerjahn F. (2012). The role of sialic acid as a modulator of the anti-inflammatory activity of IgG. *Semin. Immunopathol.* 34 443–453. 10.1007/s00281-012-0308-x 22437760

[B5] Carrano A. Hoozemans J. J. van der Vies S. M. Rozemuller A. J. van Horssen J. de Vries H. E. (2011). Amyloid Beta induces oxidative stress-mediated blood-brain barrier changes in capillary amyloid angiopathy. *Antioxid. Redox Signal.* 15 1167–1178. 10.1089/ars.2011.3895 21294650

[B6] Chang P. H. Pan Y. P. Fan C. W. Tseng W. K. Huang J. S. Wu T. H. (2016). Pretreatment serum interleukin-1beta, interleukin-6, and tumor necrosis factor-alpha levels predict the progression of colorectal cancer. *Cancer Med.* 5 426–433. 10.1002/cam4.602 26799163 PMC4799955

[B7] Chua X. Y. Chai Y. L. Chew W. S. Chong J. R. Ang H. L. Xiang P. (2020). Immunomodulatory sphingosine-1-phosphates as plasma biomarkers of Alzheimer’s disease and vascular cognitive impairment. *Alzheimers Res. Ther.* 12:122. 10.1186/s13195-020-00694-3 32998767 PMC7528375

[B8] Cruickshank J. M. (2009). Follow-up of intensive glucose control in type 2 diabetes. *N. Engl. J. Med.* 360 417–418; author reply 418. 19172673

[B9] de Jong S. E. Selman M. H. Adegnika A. A. Amoah A. S. van Riet E. Kruize Y. C. (2016). IgG1 Fc N-glycan galactosylation as a biomarker for immune activation. *Sci. Rep.* 6:28207. 10.1038/srep28207 27306703 PMC4910062

[B10] Frances A. Sandra O. Lucy U. (2016). Vascular cognitive impairment, a cardiovascular complication. *World J. Psychiatry* 6 199–207. 10.5498/wjp.v6.i2.199 27354961 PMC4919258

[B11] Gorelick P. B. Counts S. E. Nyenhuis D. (2016). Vascular cognitive impairment and dementia. *Biochim. Biophys. Acta* 1862 860–868. 26704177 10.1016/j.bbadis.2015.12.015 PMC5232167

[B12] Gregory M. A. Manuel-Apolinar L. Sanchez-Garcia S. Villa Romero A. R. de Jesus Iuit Rivera J. Basurto Acevedo L. (2019). Soluble intercellular adhesion molecule-1 (sICAM-1) as a biomarker of vascular cognitive impairment in older adults. *Dement. Geriatr. Cogn. Disord.* 47 243–253. 10.1159/000500068 31408858

[B13] Gudelj I. Lauc G. Pezer M. (2018). Immunoglobulin G glycosylation in aging and diseases. *Cell Immunol.* 333 65–79. 10.1016/j.cellimm.2018.07.009 30107893

[B14] Guoping P. Wei W. Xiaoyan L. Fangping H. Zhongqin C. Benyan L. (2015). Characteristics of the peripheral T cell immune response of patients at different stages of vascular cognitive impairment. *Immunol. Lett.* 168 120–125. 10.1016/j.imlet.2015.09.015 26433058

[B15] Hansson G. K. Robertson A. K. Soderberg-Naucler C. (2006). Inflammation and atherosclerosis. *Annu. Rev. Pathol.* 1 297–329. 18039117 10.1146/annurev.pathol.1.110304.100100

[B16] Heppner F. L. Ransohoff R. M. Becher B. (2015). Immune attack: the role of inflammation in Alzheimer disease. *Nat. Rev. Neurosci.* 16 358–372. 10.1038/nrn3880 25991443

[B17] Hermkens D. M. A. Stam O. C. G. de Wit N. M. Fontijn R. D. Jongejan A. Moerland P. D. (2019). Profiling the unique protective properties of intracranial arterial endothelial cells. *Acta Neuropathol. Commun.* 7:151. 10.1186/s40478-019-0805-4 31610812 PMC6792251

[B18] Hou H. Xu X. Sun F. Zhang X. Dong H. Wang L. (2019). Hyperuricemia is associated with immunoglobulin G N-glycosylation: a community-based study of glycan biomarkers. *OMICS* 23 660–667. 10.1089/omi.2019.0004 30835642

[B19] Iadecola C. (2013). The pathobiology of vascular dementia. *Neuron* 80 844–866. 10.1016/j.neuron.2013.10.008 24267647 PMC3842016

[B20] Jahrling J. B. Lin A. L. DeRosa N. Hussong S. A. Van Skike C. E. Girotti M. (2018). mTOR drives cerebral blood flow and memory deficits in LDLR(-/-) mice modeling atherosclerosis and vascular cognitive impairment. *J. Cereb. Blood Flow Metab.* 38 58–74. 10.1177/0271678X17705973 28511572 PMC5757441

[B21] Kiss T. Balasubramanian P. Valcarcel-Ares M. N. Tarantini S. Yabluchanskiy A. Csipo T. (2019). Nicotinamide mononucleotide (NMN) treatment attenuates oxidative stress and rescues angiogenic capacity in aged cerebromicrovascular endothelial cells: a potential mechanism for the prevention of vascular cognitive impairment. *Geroscience* 41 619–630. 10.1007/s11357-019-00074-2 31144244 PMC6885080

[B22] Kolarich D. Lepenies B. Seeberger P. H. (2012). Glycomics, glycoproteomics and the immune system. *Curr. Opin. Chem. Biol.* 16 214–220. 10.1016/j.cbpa.2011.12.006 22221852

[B23] Kristic J. Lauc G. (2017). Ubiquitous importance of protein glycosylation. *Methods Mol. Biol.* 1503 1–12. 10.1007/978-1-4939-6493-2_1 27743354

[B24] Lemmers R. F. H. Vilaj M. Urda D. Agakov F. Simurina M. Klaric L. (2017). IgG glycan patterns are associated with type 2 diabetes in independent European populations. *Biochim. Biophys. Acta Gen. Subj.* 1861 2240–2249. 10.1016/j.bbagen.2017.06.020 28668296

[B25] Li B. Li W. Li X. Zhou H. (2017). Inflammation: a novel therapeutic target/direction in atherosclerosis. *Curr. Pharm. Des.* 23 1216–1227. 10.2174/1381612822666161230142931 28034355 PMC6302344

[B26] Liu D. Chu X. Wang H. Dong J. Ge S. Q. Zhao Z. Y. (2018a). The changes of immunoglobulin G N-glycosylation in blood lipids and dyslipidaemia. *J. Transl. Med.* 16:235. 10.1186/s12967-018-1616-2 30157878 PMC6114873

[B27] Liu D. Xu X. Li Y. Zhang J. Zhang X. Li Q. (2020). Immunoglobulin G N-glycan analysis by ultra-performance liquid chromatography. *J. Vis. Exp.* 155:e60104. 10.3791/60104 32009638

[B28] Liu D. Zhao Z. Wang A. Ge S. Wang H. Zhang X. (2018b). Ischemic stroke is associated with the pro-inflammatory potential of N-glycosylated immunoglobulin G. *J. Neuroinflammation* 15:123. 10.1186/s12974-018-1161-1 29699572 PMC5921323

[B29] Liu J. Shang S. Li P. Deng M. Chen C. Jiang Y. (2017). Association between current smoking and cognitive impairment depends on age: a cross-sectional study in Xi’an, China. *Med. Clin. (Barc)* 149 203–208. 10.1016/j.medcli.2017.02.033 28416227

[B30] Liu X. Liu J. Zhao S. Zhang H. Cai W. Cai M. (2016). Interleukin-4 is essential for microglia/macrophage M2 polarization and long-term recovery after cerebral ischemia. *Stroke* 47 498–504. 10.1161/STROKEAHA.115.012079 26732561 PMC4729613

[B31] Lundstrom S. L. Yang H. Lyutvinskiy Y. Rutishauser D. Herukka S. K. Soininen H. (2014). Blood plasma IgG Fc glycans are significantly altered in Alzheimer’s disease and progressive mild cognitive impairment. *J. Alzheimers Dis.* 38 567–579. 10.3233/JAD-131088 24028868

[B32] Mallat Z. Besnard S. Duriez M. Deleuze V. Emmanuel F. Bureau M. F. (1999). Protective role of interleukin-10 in atherosclerosis. *Circ. Res.* 85 e17–e24. 10.1161/01.res.85.8.e17 10521249

[B33] Masuda K. Kubota T. Kaneko E. Iida S. Wakitani M. Kobayashi-Natsume Y. (2007). Enhanced binding affinity for FcgammaRIIIa of fucose-negative antibody is sufficient to induce maximal antibody-dependent cellular cytotoxicity. *Mol. Immunol.* 44 3122–3131. 10.1016/j.molimm.2007.02.005 17379311

[B34] Miralbell J. Lopez-Cancio E. Lopez-Oloriz J. Arenillas J. F. Barrios M. Soriano-Raya J. J. (2013). Cognitive patterns in relation to biomarkers of cerebrovascular disease and vascular risk factors. *Cerebrovasc. Dis.* 36 98–105. 10.1159/000352059 24029412

[B35] Niu Y. Wan C. Zhou B. Zhang J. Ma H. Bo Y. (2019). Breath qigong improves recognition in seniors with vascular cognitive impairment. *Altern. Ther. Health Med.* 25 20–26. 30982783

[B36] Novokmet M. Lukic E. Vuckovic F. Ethuric Z. Keser T. Rajsl K. (2014). Changes in IgG and total plasma protein glycomes in acute systemic inflammation. *Sci. Rep.* 4:4347. 10.1038/srep04347 24614541 PMC3949295

[B37] Pajares M. Rojo A. I. Manda G. Bosca L. Cuadrado A. (2020). Inflammation in Parkinson’s disease: mechanisms and therapeutic implications. *Cells* 9:1687. 10.3390/cells9071687 32674367 PMC7408280

[B38] Palmigiano A. Barone R. Sturiale L. Sanfilippo C. Bua R. O. Romeo D. A. (2016). CSF N-glycoproteomics for early diagnosis in Alzheimer’s disease. *J. Proteomics* 131 29–37. 10.1016/j.jprot.2015.10.006 26455811

[B39] Peschke B. Keller C. W. Weber P. Quast I. Lunemann J. D. (2017). Fc-Galactosylation of human immunoglobulin gamma isotypes improves C1q binding and enhances complement-dependent cytotoxicity. *Front. Immunol.* 8:646. 10.3389/fimmu.2017.00646 28634480 PMC5459932

[B40] Pinto T. C. C. Machado L. Bulgacov T. M. Rodrigues-Junior A. L. Costa M. L. G. Ximenes R. C. C. (2019). Is the montreal cognitive assessment (MoCA) screening superior to the Mini-Mental State Examination (MMSE) in the detection of mild cognitive impairment (MCI) and Alzheimer’s Disease (AD) in the elderly? *Int. Psychogeriatr.* 31 491–504. 10.1017/S1041610218001370 30426911

[B41] Pucic M. Knezevic A. Vidic J. Adamczyk B. Novokmet M. Polasek O. (2011). High throughput isolation and glycosylation analysis of IgG-variability and heritability of the IgG glycome in three isolated human populations. *Mol. Cell. Proteomics* 10:M111.010090. 10.1074/mcp.M111.010090 21653738 PMC3205872

[B42] Qin R. Yang Y. Qin W. Han J. Chen H. Zhao J. (2019). The value of serum immunoglobulin G glycome in the preoperative discrimination of peritoneal metastasis from advanced gastric cancer. *J. Cancer* 10 2811–2821. 10.7150/jca.31380 31258789 PMC6584920

[B43] Ren S. Zhang Z. Xu C. Guo L. Lu R. Sun Y. (2016). Distribution of IgG galactosylation as a promising biomarker for cancer screening in multiple cancer types. *Cell Res.* 26 963–966. 10.1038/cr.2016.83 27364686 PMC4973333

[B44] Ren Z. Q. Liu N. Zhao K. (2016). Micro RNA-19a suppresses IL-10 in peripheral B cells from patients with atherosclerosis. *Cytokine* 86 86–91. 10.1016/j.cyto.2016.07.019 27497158

[B45] Richwine A. F. Sparkman N. L. Dilger R. N. Buchanan J. B. Johnson R. W. (2009). Cognitive deficits in interleukin-10-deficient mice after peripheral injection of lipopolysaccharide. *Brain Behav. Immun.* 23 794–802. 10.1016/j.bbi.2009.02.020 19272439 PMC2881543

[B46] Ross R. (1999). Atherosclerosis–an inflammatory disease. *N. Engl. J. Med.* 340 115–126. 9887164 10.1056/NEJM199901143400207

[B47] Russell A. C. Simurina M. Garcia M. T. Novokmet M. Wang Y. Rudan I. (2017). The N-glycosylation of immunoglobulin G as a novel biomarker of Parkinson’s disease. *Glycobiology* 27 501–510. 10.1093/glycob/cwx022 28334832

[B48] Sabeti S. Schlager O. Exner M. Mlekusch W. Amighi J. Dick P. (2007). Progression of carotid stenosis detected by duplex ultrasonography predicts adverse outcomes in cardiovascular high-risk patients. *Stroke* 38 2887–2894. 10.1161/STROKEAHA.107.488387 17885257

[B49] Sebastian A. Alzain M. A. Asweto C. O. Song H. Cui L. Yu X. (2016). Glycan biomarkers for rheumatoid arthritis and its remission status in han chinese patients. *OMICS* 20 343–351. 10.1089/omi.2016.0050 27310476

[B50] Seneviratne A. N. Monaco C. (2015). Role of inflammatory cells and toll-like receptors in atherosclerosis. *Curr. Vasc. Pharmacol.* 13 146–160. 10.2174/15701611113116660160 24188491

[B51] Simonetto M. Infante M. Sacco R. L. Rundek T. Della-Morte D. (2019). A novel anti-inflammatory role of Omega-3 PUFAs in prevention and treatment of atherosclerosis and vascular cognitive impairment and dementia. *Nutrients* 11:2279. 10.3390/nu11102279 31547601 PMC6835717

[B52] Smith E. E. (2017). Clinical presentations and epidemiology of vascular dementia. *Clin. Sci. (Lond.)* 131 1059–1068. 10.1042/CS20160607 28515342

[B53] van der Flier W. M. Skoog I. Schneider J. A. Pantoni L. Mok V. Chen C. L. H. (2018). Vascular cognitive impairment. *Nat. Rev. Dis. Primers* 4:18003. 29446769 10.1038/nrdp.2018.3

[B54] Vouillarmet J. Helfre M. Maucort-Boulch D. Riche B. Thivolet C. Grange C. (2016). Carotid atherosclerosis progression and cerebrovascular events in patients with diabetes. *J. Diabetes Complications* 30 638–643. 10.1016/j.jdiacomp.2016.01.022 26969577

[B55] Vuckovic F. Kristic J. Gudelj I. Teruel M. Keser T. Pezer M. (2015). Association of systemic lupus erythematosus with decreased immunosuppressive potential of the IgG glycome. *Arthritis Rheumatol.* 67 2978–2989. 10.1002/art.39273 26200652 PMC4626261

[B56] Wang S. Xu L. Jonas J. B. You Q. S. Wang Y. X. Yang H. (2011). Prevalence and associated factors of dyslipidemia in the adult Chinese population. *PLoS One* 6:e17326. 10.1371/journal.pone.0017326 21423741 PMC3053360

[B57] Wang X. X. Zhang B. Xia R. Jia Q. Y. (2020). Inflammation, apoptosis and autophagy as critical players in vascular dementia. *Eur. Rev. Med. Pharmacol. Sci.* 24 9601–9614. 10.26355/eurrev_202009_23048 33015803

[B58] Wang X. Li W. Song F. Wang L. Fu Q. Cao S. (2018). Carotid atherosclerosis detected by ultrasonography: a national cross-sectional study. *J. Am. Heart Assoc.* 7:e008701. 10.1161/JAHA.118.008701 29622590 PMC6015437

[B59] Wang Y. Klaric L. Yu X. Thaqi K. Dong J. Novokmet M. (2016). The association between glycosylation of immunoglobulin G and hypertension: a multiple ethnic cross-sectional Study. *Medicine (Baltimore)* 95:e3379. 10.1097/MD.0000000000003379 27124023 PMC4998686

[B60] Wu Z. Pan H. Liu D. Zhou D. Tao L. Zhang J. (2021). Variation of IgG N-linked glycosylation profile in diabetic retinopathy. *J. Diabetes* 13 672–680. 10.1111/1753-0407.13160 33491329

[B61] Yu X. Wang Y. Kristic J. Dong J. Chu X. Ge S. (2016). Profiling IgG N-glycans as potential biomarker of chronological and biological ages: a community-based study in a Han Chinese population. *Medicine (Baltimore)* 95:e4112. 10.1097/MD.0000000000004112 27428197 PMC4956791

[B62] Zhu Y. Xian X. Wang Z. Bi Y. Chen Q. Han X. (2018). Research Progress on the Relationship between Atherosclerosis and Inflammation. *Biomolecules* 8:80. 10.3390/biom8030080 30142970 PMC6163673

[B63] Zou G. Ochiai H. Huang W. Yang Q. Li C. Wang L. X. (2011). Chemoenzymatic synthesis and Fcgamma receptor binding of homogeneous glycoforms of antibody Fc domain. Presence of a bisecting sugar moiety enhances the affinity of Fc to FcgammaIIIa receptor. *J. Am. Chem. Soc.* 133 18975–18991. 10.1021/ja208390n 22004528 PMC3218234

