# Supplementary material for: Association Between Immunoglobulin G N-glycosylation and Vascular Cognitive Impairment in a Sample With Atherosclerosis: A Case-Control Study
Source: Front Aging Neurosci. 2022 Feb 10;14:823468. doi: 10.3389/fnagi.2022.823468 (PMC8868374; doi:10.3389/fnagi.2022.823468)
Supplement: Supplementary file 1 [file Data_Sheet_1.docx]

**Supplementary**

**
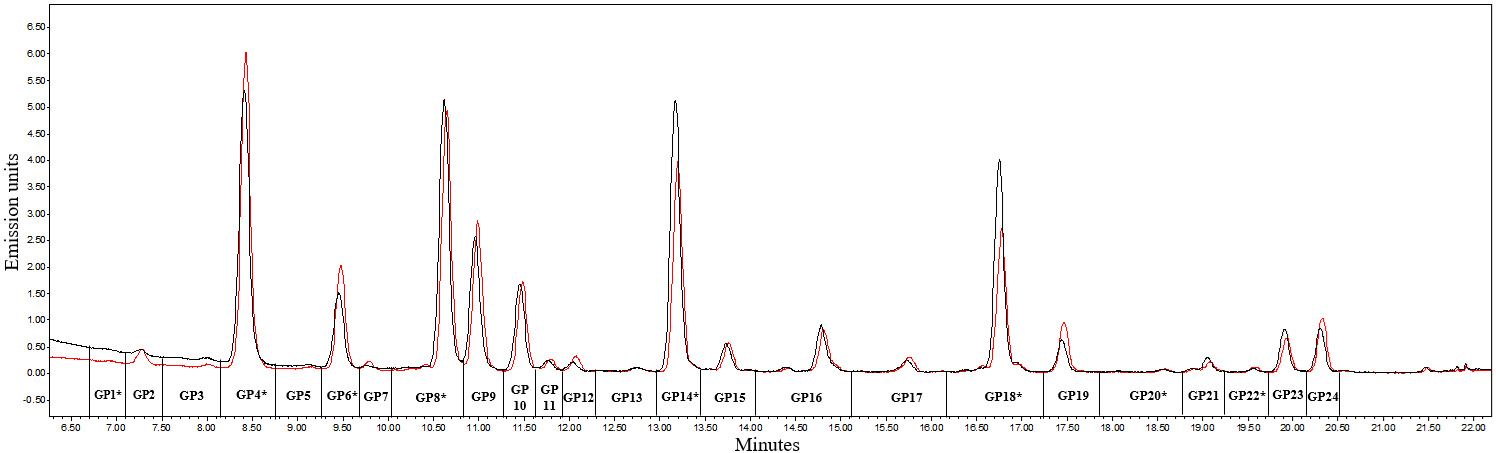
Figure S1.** A chromatogram showing differential *N*-glycan peaks of an individual with or without VCI. *There is a statistical difference between the case group and the control group (*P*<0.05, *q*<0.05). Red represents the distribution of *N*-glycan peaks of a case; black represents the distribution of *N*-glycan peaks of a control. This figure shows the difference in initial glycans between individuals with or without VCI, and it cannot fully reflect all the differences between the initial glycans in the overall case group and the control group. GP, glycan peak; VCI, vascular cognitive impairment.

**
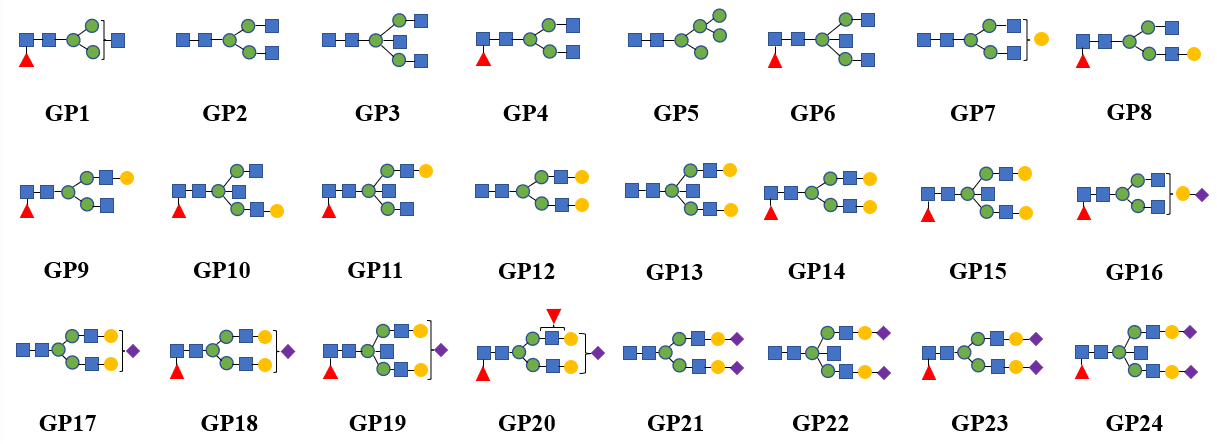
**

**Figure S2. Structures of the initial IgG glycome.** Blue squares represent bisecting GlcNAc; green circles represent mannose; red triangles represent fucose; yellow circles represent galactose; purple diamonds represent sialic acid. GP, glycan peak; GlcNAc, N-acetylglucosamine; IgG, immunoglobulin G.

**Table S1. The levels of initial glycans from the control group and case group.**

| Glycans | Cases | Controls | Z | *P* | *q* | |
| --- | --- | --- | --- | --- | --- | --- |
|  | Median (*P*_25_–*P*_75_) | Median (*P*_25_–*P*_75_) |  |  |  |  |
| GP1 | 0.38 (0.22-0.70) | 0.24 (0.13-0.40) | 4.607 | 4.00E-06* | 2.30E-05^#^ | |
| GP2 | 0.44 (0.24-0.77) | 0.39 (0.21-0.73) | 0.933 | 3.51E-01 | 5.38E-01 | |
| GP4 | 24.25 (20.79-27.60) | 22.30 (18.74-26.34) | 2.547 | 1.09E-02* | 3.12E-02^#^ | |
| GP5 | 0.14 (0.08-0.22) | 0.12 (0.06-0.23) | 0.624 | 5.32E-01 | 6.80E-01 | |
| GP6 | 5.64 (4.76-6.46) | 4.67 (3.72-5.82) | 5.392 | 6.97E-08* | 5.34E-07^#^ | |
| GP7 | 0.50 (0.16-0.78) | 0.38 (0.13-0.89) | 0.587 | 5.57E-01 | 6.75E-01 | |
| GP8 | 16.17 (13.53-18.94) | 14.92 (12.75-17.29) | 3.317 | 9.10E-04* | 3.49E-03^#^ | |
| GP9 | 7.33 (5.36-9.55) | 7.40 (5.66-9.54) | 0.456 | 6.48E-01 | 7.45E-01 | |
| GP10 | 7.14 (4.52-9.09) | 7.33 (4.48-9.26) | 0.083 | 9.34E-01 | 9.67E-01 | |
| GP11 | 0.46 (0.33-0.69) | 0.56 (0.35-0.73) | 1.937 | 5.28E-02 | 1.21E-01 | |
| GP12 | 0.63 (0.48-0.85) | 0.61 (0.43-0.78) | 0.625 | 5.32E-01 | 7.20E-01 | |
| GP13 | 0.95 (0.57-1.24) | 1.10 (0.53-1.65) | 2.009 | 4.46E-02* | 1.14E-01 | |
| GP14 | 10.97 (9.42-13.08) | 13.18 (10.83-15.29) | 5.452 | 4.98E-08* | 5.73E-07^#^ | |
| GP15 | 1.54 (1.12-1.88) | 1.38 (1.06-1.76) | 1.923 | 5.45E-02 | 1.14E-01 | |
| GP16 | 2.87 (2.32-3.49) | 2.86 (2.43-3.43) | 0.694 | 4.88E-01 | 7.01E-01 | |
| GP17 | 1.39 (0.62-2.27) | 1.54 (0.73-2.37) | 1.173 | 2.41E-01 | 3.96E-01 | |
| GP18 | 8.28 (6.03-9.72) | 8.91 (7.26-10.79) | 3.565 | 3.64E-04* | 1.67E-03^#^ | |
| GP19 | 2.25 (1.81-2.71) | 2.19 (1.67-2.64) | 1.398 | 1.62E-01 | 2.87E-01 | |
| GP20 | 0.35 (0.23-0.72) | 0.52 (0.29-0.84) | 2.730 | 6.34E-03* | 2.08E-02^#^ | |
| GP21 | 1.18 (0.57-1.82) | 1.19 (0.53-1.71) | 0.041 | 9.67E-01 | 9.67E-01 | |
| GP22 | 1.46 (1.00-1.80) | 0.93 (0.59-1.40) | 6.646 | 3.01E-11* | 6.91E-10^#^ | |
| GP23 | 1.51 (1.12-1.78) | 1.52 (1.11-2.15) | 1.914 | 5.56E-02 | 1.07E-01 | |
| GP24 | 1.25 (0.92-2.27) | 1.31 (0.86-1.97) | 0.386 | 6.99E-01 | 7.66E-01 | |
| *Statistically significant, *P*＜0.05; ^#^Significant after correction using FDR, *q*<0.05. *P* values were calculated by the Wilcoxon rank-sum test. FDR, false discovery rate; GP, glycan peak; *P*_25_, the 25th percentile; *P*_75_, the 75th percentile. | | | | | |  |

**Table S2. The levels of glycans derived from the control group and case group.**

| Derived traits | Cases | Controls | Z | *P* | *q* |
| --- | --- | --- | --- | --- | --- |
|  | Median (*P*_25_–*P*_75_) | Median (*P*_25_–*P*_75_) |  |  |  |
| GPN | 77.50 (74.72-79.59) | 75.89 (73.80-79.07) | 2.851 | 4.36E-03* | 8.24E-03^#^ |
| S_total_ | 20.50 (18.49-22.38) | 21.37 (18.77-23.99) | 2.500 | 1.24E-02* | 1.76E-02^#^ |
| S1 | 14.76 (12.57-16.92) | 16.10 (13.88-17.83) | 3.734 | 1.89E-04* | 5.36E-04^#^ |
| S2 | 5.80 (4.79-6.71) | 5.14 (4.40-6.32) | 2.984 | 2.85E-03* | 6.05E-03^#^ |
| G0 | 31.16 (27.66-34.42) | 28.65 (24.00-32.63) | 4.079 | 4.50E-05* | 1.53E-04^#^ |
| G1 | 32.49 (29.46-34.61) | 31.27 (28.18-33.33) | 3.082 | 2.06E-03* | 5.00E-03^#^ |
| G2 | 14.18 (12.23-16.20) | 16.38 (13.95-18.92) | 6.030 | 1.64E-09* | 2.79E-08^#^ |
| F | 90.35 (88.24-92.61) | 90.24 (87.97-92.88) | 0.366 | 7.14E-01 | 7.14E-01 |
| FN | 96.24 (95.55-96.92) | 96.07 (94.46-97.24) | 1.101 | 2.71E-01 | 3.07E-01 |
| FS | 78.97 (73.90-83.80) | 80.54 (76.39-86.23) | 2.348 | 1.89E-02* | 2.29E-02^#^ |
| B | 20.93 (18.67-23.75) | 19.95 (17.65-22.34) | 2.418 | 1.56E-02* | 2.04E-02^#^ |
| BN | 18.13 (14.51-21.69) | 18.89 (14.44-21.24) | 0.479 | 6.32E-01 | 6.71E-01 |
| BS | 25.68 (22.41-30.60) | 21.93 (17.94-25.47) | 5.964 | 2.46E-09* | 2.09E-08^#^ |
| FG0 | 24.25 (20.79-27.60) | 22.30 (18.74-26.34) | 2.547 | 1.09E-02* | 1.68E-02^#^ |
| FG1 | 23.33 (19.92-27.85) | 22.03 (19.92-25.10) | 2.641 | 8.27E-03* | 1.41E-02^#^ |
| FG2 | 10.97 (9.42-13.08) | 13.18 (10.83-15.29) | 5.452 | 4.98E-08* | 2.82E-07^#^ |
| aGal/Gal ratio | 51.77 (43.77-59.99) | 44.61 (34.91-56.00) | 4.178 | 2.90E-05* | 1.23E-04^#^ |
| *Statistically significant, *P*＜0.05; ^#^Significant after correction using FDR, *q*<0.05. *P* values were calculated by the Wilcoxon rank-sum test. B, bisecting GlcNAc; F, core fucose; FDR, false discovery rate; G, galactose; N, neutral glycans; *P*_25_, the 25th percentile; *P*_75_, the 75th percentile; S, sialic acid. | | | | | |

**Table S3. The canonical sets in the results of canonical correlation analysis.**

| **canonical sets** | ***r*** | ***F*** | ***P*** |
| --- | --- | --- | --- |
| **1** | **0.272** | **1.965** | **0.004** |
| **2** | **0.224** | **1.556** | **0.087** |
| **3** | **0.124** | **0.836** | **0.542** |

**
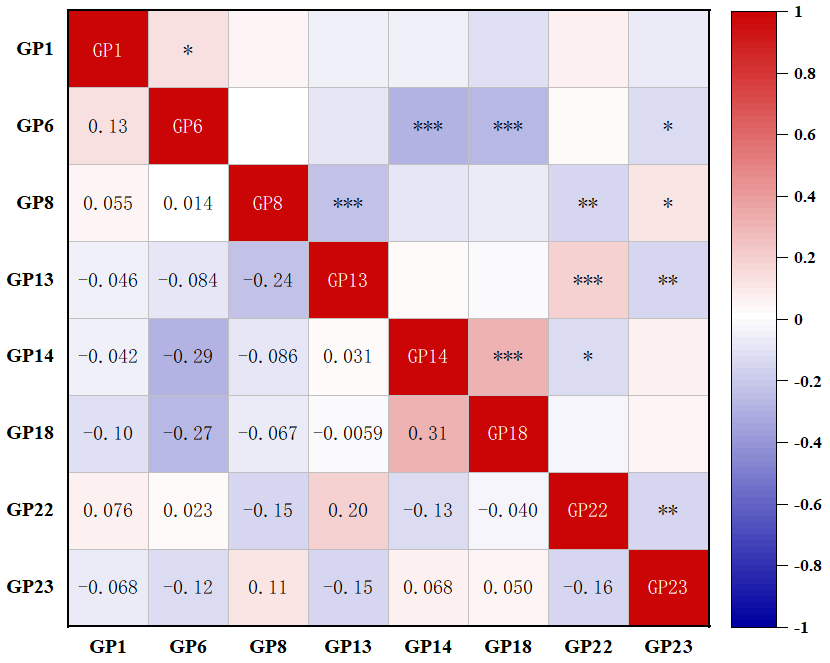
**

**Figure S3. The correlation coefficient of independent glycans obtained by Spearman correlation analysis.** The positive correlations are represented by red, while negative correlations are represented by blue. ^*^*P*＜0.05; ^**^*P*＜0.01; ^***^*P*＜0.001. GP, glycan peak.

**Table S4. A stepwise multivariate logistic regression analysis of the association of Glycans with VCI.**

| Glycans | *β* | *SE* | Wald | OR | 95% CI of OR | | *P* |
| --- | --- | --- | --- | --- | --- | --- | --- |
|  |  |  |  |  | LCI | UCI |  |
| GP1 | 0.849 | 0.360 | 5.560 | 2.337 | 1.154 | 4.732 | 0.018 |
| GP6 | 0.330 | 0.093 | 12.640 | 1.390 | 1.159 | 1.668 | ＜0.001 |
| GP8 | 0.110 | 0.036 | 9.178 | 1.116 | 1.040 | 1.198 | 0.002 |
| GP13 | -0.568 | 0.209 | 7.350 | 0.567 | 0.376 | 0.854 | 0.007 |
| GP14 | -0.141 | 0.046 | 9.356 | 0.869 | 0.794 | 0.951 | 0.002 |
| GP22 | 0.971 | 0.197 | 24.163 | 2.639 | 1.792 | 3.887 | ＜0.001 |
| GP23 | -0.603 | 0.219 | 7.603 | 0.547 | 0.356 | 0.840 | 0.006 |
| Education | -0.654 | 0.255 | 6.580 | 0.520 | 0.315 | 0.857 | 0.010 |
| Income | -0.546 | 0.258 | 4.498 | 0.579 | 0.350 | 0.959 | 0.034 |
| Constant | 0.425 | 1.400 | 0.092 | 1.529 | — | — | 0.762 |
| *R*^2^=0.410. GP1, GP6, GP8, GP13, GP14, GP18, GP22, and GP23 were included in the model (adjusted for age, BMI, education, income, smoking, drinking, salt intake habit, hypertension, hyperlipidaemia, and diabetes mellitus). *β*, regression coefficient; BMI, body mass index; CI, confidence interval; GP, glycan peak; LCI, lower confidence interval; OR, odds ratio; *SE*, standard error; UCI, upper confidence interval; VCI, vascular cognitive impairment. | | | | | | | |

**Table S5. A stepwise multivariate logistic regression analysis of the association of inflammation factors with VCI.**

| Inflammation  factors | *β* | *SE* | Wald | OR | 95% CI of OR | | *P* |
| --- | --- | --- | --- | --- | --- | --- | --- |
|  |  |  |  |  | LCI | UCI |  |
| TNF-α | 0.127 | 0.023 | 29.726 | 1.135 | 1.085 | 1.189 | ＜0.001 |
| IL-6 | 0.118 | 0.052 | 5.085 | 1.126 | 1.016 | 1.248 | 0.024 |
| IL-10 | -0.021 | 0.004 | 31.016 | 0.980 | 0.973 | 0.987 | ＜0.001 |
| Education | -0.626 | 0.233 | 7.246 | 0.535 | 0.339 | 0.844 | 0.007 |
| Hypertension | 0.550 | 0.269 | 4.182 | 1.734 | 1.023 | 2.939 | 0.041 |
| Constant | 0.569 | 0.909 | 0.393 | 1.767 | — | — | 0.531 |
| *R*^2^=0.333. TNF-α, IL-6, IL-10, IL-4, and hs-CRP were included in the model (adjusted for age, BMI, education, income, smoking, drinking, salt intake habit, hypertension, hyperlipidaemia, and diabetes mellitus). *β*, regression coefficient; BMI, body mass index; CI, confidence interval; hs-CRP, high-sensitivity C-reactive protein; IL-4, interleukin-4; IL-6, interleukin-6; IL-10, interleukin-10; LCI, lower confidence interval; OR, odds ratio; *SE*, standard error; TNF-α, tumor necrosis factor-alpha; UCI, upper confidence interval; VCI, vascular cognitive impairment. | | | | | | | |
